# Surface micro-patterned drug-coated balloon enhances early biological efficacy in a porcine coronary in-stent restenosis model

**DOI:** 10.1371/journal.pone.0359232

**Published:** 2026-09-25

**Authors:** Seunghwan Kim, Seul-Gee Lee, Joonoh Park, Jino Park, Dong-Kie Kim, Ki-Hun Kim, Doo-Il Kim, Se-Il Park, Ok-Hee Jeon, Yong-Jun Lee, Oh-Hyun Lee, Dong-Eon Kim, WonHyoung Ryu, Jung-Sun Kim

**Affiliations:** 1 Division of Cardiology, Haeundae Paik Hospital, Inje University College of Medicine, Busan, Korea; 2 Yonsei University College of Medicine, Seoul, Korea; 3 Integrative Research Center for Cerebrovascular and Cardiovascular Diseases, Division of Cardiology, Yonsei University College of Medicine, Seoul, Korea; 4 Cardiovascular Product Evaluation Center, Yonsei University College of Medicine, Seoul, Korea; 5 Division of Cardiology, Severance Cardiovascular Hospital, Yonsei University College of Medicine, Seoul, Korea; 6 Division of Cardiology, Yongin Severance Hospital, Yonsei University College of Medicine, Gyeonggi-do, Korea; 7 Division of Cardiology, Soonchunhyang University Bucheon Hospital, Soonchunhyang University College of Medicine, Bucheon, Korea; 8 Department of Mechanical Engineering, Yonsei University, Seoul, Korea; IPSurface Canada Inc, CANADA

## Abstract

**Background:**

Drug-coated balloon (DCB) therapy for in-stent restenosis (ISR) relies on efficient drug transfer during balloon–artery contact. Surface micro-patterning has been proposed to enhance transfer efficiency without altering the antiproliferative drug. This study evaluated a linear micro-patterned DCB (LMDCB) compared with a comparator paclitaxel-coated DCB in a porcine coronary ISR model.

**Methods:**

ISR **was induced by bare-metal stent implantation followed by a 4-week maturation period.** In two prespecified cohorts (1 month: 10 animals, 20 segments; 3 months: 4 animals, 8 segments), paired ISR segments within each animal were treated with LMDCB or a comparator paclitaxel-coated DCB (3.0 µg/mm^2^). Endpoints included quantitative coronary angiography (QCA), optical coherence tomography (OCT), and histology.

**Results:**

At 1 month, LMDCB demonstrated a larger minimum lumen diameter (2.3 ± 0.2 mm vs 1.9 ± 0.4 mm; p = 0.019) and lower percent diameter stenosis (13.3 ± 4.8% vs 23.7 ± 9.3%; p = 0.003). OCT showed lower mean neointimal thickness (0.2 ± 0.1 mm vs 0.3 ± 0.1 mm; p = 0.008). Histology showed lower neointimal area (2.4 ± 0.7 mm^2^ vs 2.9 ± 1.1 mm^2^; p = 0.011) and percent area stenosis (36.6 ± 10.4% vs 44.4 ± 17.8%; p = 0.013), with comparable vascular healing indices. In the independent 3-month cohort, QCA and OCT findings were similar between groups. Exploratory histology in a limited subset suggested a larger lumen and lower percent area stenosis with LMDCB.

**Conclusions:**

In this porcine coronary ISR model, LMDCB was associated with reduced neointimal proliferation and improved luminal dimensions at 1 month compared with a comparator paclitaxel-coated DCB, without impaired vascular healing. In a separate 3-month cohort, imaging findings were comparable between devices, while histology remained exploratory.

## Introduction

In-stent restenosis (ISR) remains a clinically relevant limitation of percutaneous coronary intervention despite advances in stent design and implantation techniques, and it continues to necessitate repeat revascularisation in a substantial proportion of patients [1]. Drug-coated balloon (DCB) therapy has emerged as an established treatment strategy for ISR by delivering an antiproliferative drug during brief balloon inflation while avoiding additional metallic layers, thereby preserving a *leave-nothing-behind* strategy in previously stented segments [2–4].

The therapeutic effectiveness of DCBs depends on efficient transfer of the antiproliferative drug from the balloon surface to the arterial wall during the short period of balloon–artery contact. However, ISR lesions often exhibit heterogeneous neointimal morphology and irregular luminal geometry within the stented segment, which may compromise uniform balloon apposition and limit local drug deposition despite an adequate nominal dose [1,5,6]. These considerations suggest that treatment outcomes may be influenced not only by the pharmacological agent but also by the efficiency of device–tissue interaction during balloon inflation.

Consistent with this concept, comparative studies in coronary ISR have not identified a uniformly superior antiproliferative drug across current DCB platforms, and device-specific performance may also vary among balloons delivering the same drug but using different coating technologies or excipient formulations. Together, these observations support the view that coating–tissue interaction and delivery mechanics are important determinants of effective drug transfer and biological response beyond nominal drug dose alone [7–9].

Device-based engineering strategies aimed at optimising the balloon–artery interface may therefore represent a complementary approach to improving DCB performance. Linear micro-patterned drug-coated balloon (LMDCB) platforms incorporate longitudinal micro-architectural surface structures designed to increase focal contact engagement during balloon inflation and thereby facilitate drug transfer to the vessel wall. Prior preclinical work with an earlier prototype of this platform reported increased contact pressure and greater short-term paclitaxel retention in rabbit iliac arteries, together with favourable imaging findings in a limited minipig coronary ISR model [10].

The present study was designed to evaluate whether a linear micro-patterned DCB improves biological efficacy compared with a comparator paclitaxel-coated DCB in a porcine coronary ISR model**.** Using a paired within-animal design, we conducted a staged translational preclinical study with two prespecified, independently analysed follow-up cohorts. A 1-month cohort was used to evaluate the early biological response to treatment, while a separate 3-month cohort was included to provide supportive mid-term context regarding neointimal response and vascular healing.

## Methods

### Study design and ethical approval

This staged translational preclinical study compared an investigational LMDCB with a comparator paclitaxel-coated DCB in a porcine coronary ISR model using a paired within-animal design. The 1-month and 3-month assessments were conducted sequentially in separately procured animal cohorts as part of a staged preclinical development programme. Separate cohorts were required because histological assessment at each follow-up time point involved terminal euthanasia and tissue harvesting; therefore, the same animals could not contribute histological data at both time points. The study was designed to evaluate the early biological response and vascular healing at 1 month and to provide supportive information regarding the vascular response and healing at a later follow-up time point. Accordingly, separate cohorts underwent terminal follow-up at 1 month (n = 10 animals) or 3 months (n = 4 animals) after DCB treatment. In each animal, ISR was created in two coronary arteries. After ISR confirmation at 4 weeks post-stenting, one vessel was treated with LMDCB and the paired vessel with the comparator DCB. The LMDCB and comparator DCB were assigned to the two paired coronary segments in a 1:1 ratio according to a randomisation schedule generated using a random-number table before the interventional procedures. Allocation was concealed from the interventional operator until the paired target segments had been selected and baseline angiographic and OCT assessments had been completed. The interventional operator became aware of device assignment during device preparation and was therefore not blinded during DCB deployment. Between-device comparisons were performed using paired observations within the same animal. Analyses were performed separately for the 1-month and 3-month cohorts, and no pooled analysis across follow-up cohorts was conducted. The study was approved by the Animal Ethics Committee and the Institutional Animal Care and Use Committee (IACUC) at the Cardiovascular Product Evaluation Center, Yonsei University College of Medicine (CPEC-IACUC-161007 and CPEC-IACUC-181003). Animal care complied with the Animal Welfare Act and the “Principles of Laboratory Animal Care” (NIH Publication No. 85−23, revised 1996). The study was conducted in accordance with the ARRIVE guidelines for reporting animal research.

### Animals and peri-procedural management

Initially, 17 miniature pigs (approximately 6 months old; 35–40 kg) were used in this study. Miniature pigs were selected for their anatomical similarity to human coronary anatomy and adequate size for catheterisation. Aspirin (10 mg/day) and clopidogrel (75 mg/day) were administered throughout the study period. Animals were sedated with intramuscular atropine (0.05 mg/kg), tramadol (5 mg/kg), and Baytril (5 mg/kg). Induction anaesthesia included intravenous alfaxan (0.2 mg/kg) and rompun (0.05 mg/kg), and anaesthesia was maintained with 1.5% isoflurane in oxygen. Coronary access was obtained via the carotid artery. Unfractionated heparin (200 U/kg) was administered intraprocedurally to maintain an activated clotting time of approximately 250 seconds. Physiological monitoring was performed using the Mac-Lab system (GE, USA). All procedures were performed by researchers trained in animal care and handling. Postoperative analgesia was provided as needed during the recovery period.

During the study period, the animals were housed under controlled conditions (12-hour light/dark cycle) with free access to food and water, and their health and behaviour were monitored daily. Humane endpoints were defined prior to study initiation, and criteria for early euthanasia included unexpected signs of severe distress, including laboured breathing, unresponsiveness, or prolonged anorexia or adipsia. If these criteria were met, the animal was to be euthanised immediately to prevent further suffering. Before ISR induction, 12 animals were allocated to the 1-month cohort and 5 to the 3-month cohort. Two animals in the 1-month cohort and one in the 3-month cohort died unexpectedly during or immediately after balloon overstretch injury and bare-metal stent implantation. These animals were excluded from follow-up. These deaths occurred approximately 4 weeks before DCB treatment and therefore before exposure to either the investigational LMDCB or the comparator DCB. The remaining 14 miniature pigs (10 in the 1-month cohort and 4 in the 3-month cohort) completed the planned follow-up without meeting the predefined criteria for early euthanasia.

### ISR induction protocol

ISR was induced in two coronary arteries per animal (right coronary artery, left anterior descending artery, or left circumflex artery) by balloon overstretch injury followed by bare-metal stent (BMS) implantation. Balloon overstretch injury was performed using a 1.2:1.0 balloon-to-artery diameter ratio for 60 seconds. Subsequently, an identical 3.0 mm × 18 mm BMS (Genoss Co., Ltd., Seoul, Korea) was implanted in each target vessel using an approximately 1.2 × reference vessel diameter strategy; stent balloon inflation was repeated twice for 30 seconds each. Accordingly, lesion length was standardised by the implanted 18-mm stent and was therefore not analysed as an independent lesion characteristic. ISR was allowed to develop for 4 weeks and was confirmed by coronary angiography and OCT.

### DCB treatment protocol and follow-up

**Fig 1:**
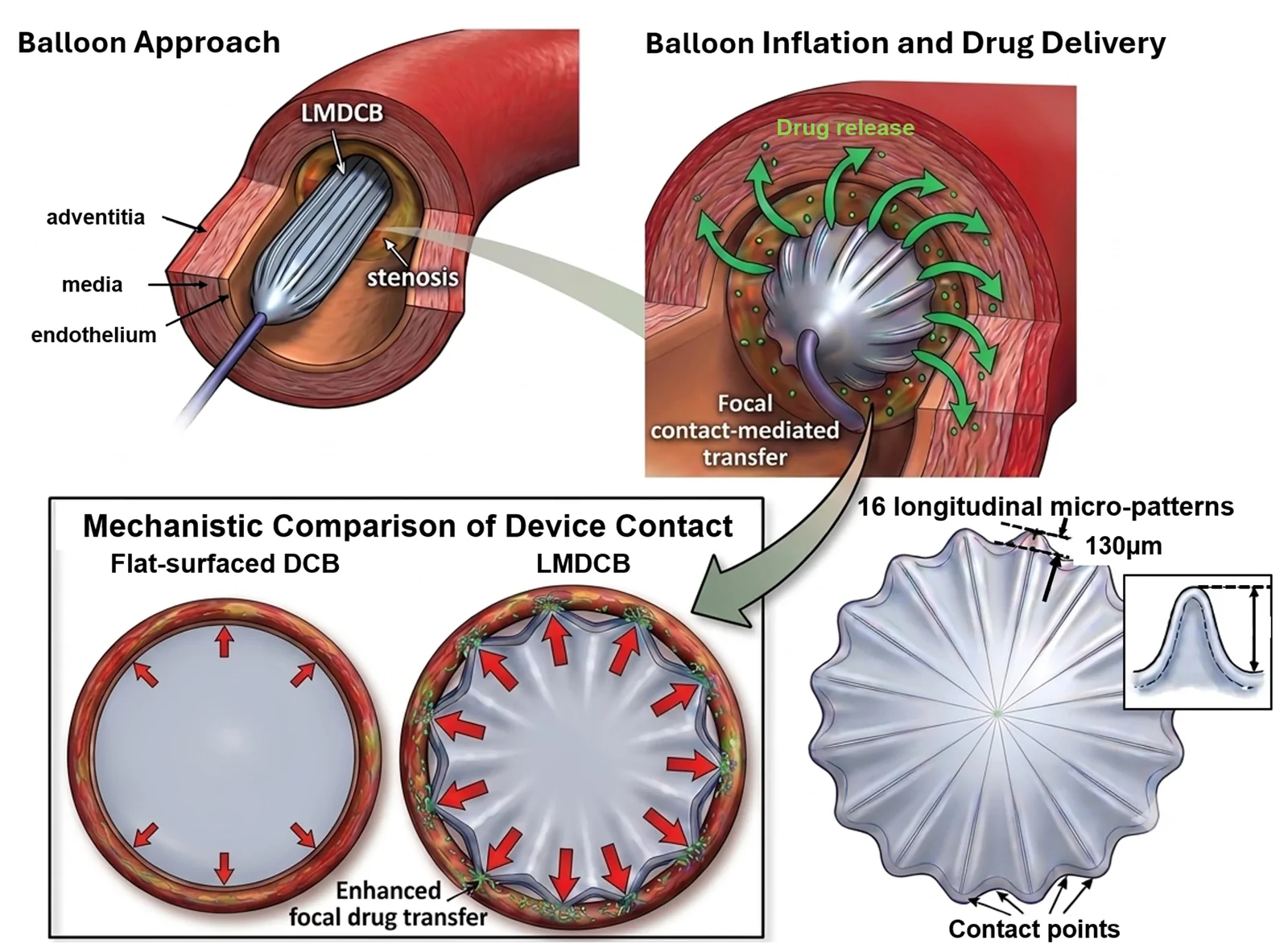
Schematic and cross-sectional comparison of a flat-surfaced drug-coated balloon and the linear micro-patterned drug-coated balloon.

DCB treatment was performed in both ISR vessels within each animal (LMDCB in one vessel and comparator DCB in the other). A 3.0 × 20 mm balloon was used for all treated segments in both the investigational LMDCB and comparator DCB groups, corresponding to an approximate balloon-to-reference vessel diameter ratio of 1.2 based on the angiographically measured reference vessel diameter. Balloon inflation was maintained for 60 seconds with a single inflation per vessel. A 60-second inflation duration has also been used in a randomised clinical study of coronary DCB treatment for ISR [3]. The same balloon dimensions, sizing approach, and single-inflation protocol were applied to both devices to preserve internal comparability. However, inflation duration and the use of repeat inflation may vary in clinical practice according to lesion and procedural characteristics. The investigational LMDCB is a paclitaxel-coated balloon (3.0 µg/mm²) featuring 16 conformal longitudinal ridge-shaped linear micro-patterns (height, 130 µm), as previously described [10]. These surface structures are designed to generate focal contact points at the balloon–artery interface during inflation, which may increase local contact pressure and facilitate contact-mediated paclitaxel transfer to the vessel wall. The comparator device was Pantera Lux (Biotronik, Bülach, Switzerland), a paclitaxel-coated DCB with the same nominal drug dose density of 3.0 µg/mm² and a smooth, non-micropatterned balloon surface. Pantera Lux was selected as the active comparator because it is a clinically used DCB that delivers the same antiproliferative drug at the same nominal dose density as the investigational LMDCB, thereby allowing comparison while matching drug identity and nominal dose density. A schematic and cross-sectional comparison of a flat-surfaced DCB and the LMDCB, together with the proposed focal contact-mediated drug-transfer mechanism, is shown in Fig 1.

A flat-surfaced DCB is depicted as having a broad balloon–artery contact interface, whereas the LMDCB incorporates 16 longitudinal ridge-shaped micro-patterns, approximately 130 µm in height, designed to generate focal contact points during inflation. These focal contact points may increase local contact pressure and facilitate contact-mediated drug transfer to the vessel wall. The proposed mechanism is based on the device architecture and prior mechanistic findings reported by Lee et al. [10] and was not directly evaluated in the present study. The schematic was newly created for the present study and is not to scale. DCB, drug-coated balloon; LMDCB, linear micro-patterned drug-coated balloon.

The coating chemistry and key device characteristics of the LMDCB and comparator DCB are summarised in Table 1. Each device was inflated at its respective manufacturer-specified nominal pressure: 8 atm for the LMDCB and 7 atm for the comparator DCB.

**Table 1:** Coating chemistry and key device characteristics of the LMDCB and comparator DCB.

Parameter	LMDCB	Comparator DCB
Manufacturer	Genoss Co., Ltd., Seoul, Korea	Biotronik, Bülach, Switzerland
Balloon diameter × length	3.0 × 20 mm	3.0 × 20 mm
Drug	Paclitaxel	Paclitaxel
Drug dose	3.0 µg/mm²	3.0 µg/mm²
Coating matrix/excipient	Shellac-based natural resin	BTHC-based matrix
Balloon surface architecture	Linear micro-patterned surface with 16 conformal linear micro-patterns, 130 µm in height	Smooth, non-micropatterned surface
Nominal pressure	8 atm	7 atm
Inflation duration	60 seconds	60 seconds

DCB, drug-coated balloon; LMDCB, linear micro-patterned drug-coated balloon; BTHC, butyryl-tri-hexyl citrate. Device specifications, including balloon diameter and length, drug dose, coating matrix/excipient, and nominal pressure, were obtained from manufacturer-provided information. The surface architecture and mechanistic characterisation of an earlier prototype of the linear micro-patterned platform were previously reported [10].

Procedural device-related complications, including balloon rupture, coating detachment, shaft fracture, or incomplete balloon expansion, were prospectively recorded during each intervention. Animals continued dual antiplatelet therapy and were followed to terminal assessment at 1 month or 3 months after DCB treatment. At the terminal time point, animals were euthanised under deep anaesthesia via intravenous potassium chloride overdose.

### Quantitative coronary angiography (QCA)

Coronary angiography was performed during ISR induction, including after stent implantation, and at the time of DCB treatment and follow-up. For QCA analysis, angiograms obtained immediately before DCB treatment (4 weeks after stent implantation), immediately after DCB treatment, and at terminal follow-up were analysed using the CAAS system (Pie Medical Instruments, the Netherlands). Minimum lumen diameter (MLD) and reference diameter (RD) were determined at end-diastole in matched projections using the software’s automated edge-detection algorithm. The RD was defined as the reconstructed diameter at the location of the MLD, as determined by the software’s interpolation algorithm. Percent diameter stenosis (%DS) was calculated as [(RD − MLD)/RD] × 100. Acute gain was defined as the MLD immediately after DCB treatment minus the MLD immediately before DCB treatment, and late lumen loss was defined as the MLD immediately after DCB treatment minus the MLD at follow-up. QCA analyses were performed by investigators blinded to treatment allocation.

### Optical coherence tomography (OCT)

OCT imaging was acquired using the C7-XR system (LightLab Imaging Inc., St. Jude Medical, USA). Automated pullback was performed at 20 mm/s with an image acquisition rate of 100 frames/s. During imaging, the coronary lumen was cleared by continuous flushing of contrast media through the guiding catheter at 4 mL/s. Cross-sections were analysed at 1-mm intervals along the 20-mm stented segment. OCT-derived parameters included stent area, lumen area, neointimal area (stent area − lumen area), neointimal thickness, and percent area stenosis (%AS), calculated as [(stent area − lumen area)/stent area] × 100. All OCT analyses were performed offline by an experienced analyst blinded to treatment allocation.

### Histology, histomorphometry, and semi-quantitative healing assessment

Following euthanasia, the harvested heart was perfusion-fixed by connecting a fixative tube to the aorta and applying 10% formalin at 100–130 mmHg for 24 hours. The stented arteries were embedded in resin (Technovit 7200 VLC; Kulzer, Wehrheim, Germany). Embedded blocks were cut into proximal, mid, and distal cross-sections (220 ± 20 µm) using a diamond cutting system (EXAKT 300 CP, Germany), mounted on glass slides using an adhesive press system (EXAKT 402, Germany), and ground to a final thickness of 35 ± 5 µm (EXAKT 400CS, Germany). Sections were stained with haematoxylin and eosin (H&E) and Masson's trichrome and examined by light microscopy. Sections with severe artefacts or incomplete stent strut representation were excluded from analysis. Quantitative histomorphometric analysis was performed to measure lumen area, internal elastic lamina (IEL) area, and external elastic lamina (EEL) area. Neointimal area was calculated as IEL area − lumen area, and percent area stenosis (%AS) as [1 − (lumen area/IEL area)] × 100 for each section. Semi-quantitative histological scoring was performed according to established grading systems: arterial injury was assessed using the Schwartz score (0–3) determined by the depth of injury relative to the IEL and media [11]; inflammation and endothelialisation scores were assessed using published criteria [12]; and fibrin deposition was scored from 0 to 3 as previously described [13].

### Study endpoints

Analyses were conducted separately for the 1-month and 3-month cohorts. A single primary efficacy endpoint was not explicitly specified in the original study protocol. Accordingly, for interpretive purposes, OCT-derived mean neointimal thickness at 1 month was designated post hoc as the primary efficacy endpoint. Other 1-month QCA and OCT measures, histomorphometric outcomes, and semi-quantitative vascular-healing measures were considered secondary or supportive outcomes. All corresponding findings from the independent 3-month cohort were considered exploratory because of the smaller sample size, particularly for histological assessment. All endpoint analyses were performed by investigators blinded to treatment allocation.

### Statistical analysis

Statistical analyses were performed using R, version 4.5.2 (R Foundation for Statistical Computing, Vienna, Austria). No formal a priori sample size calculation was performed, and sample size was informed by previous data from porcine ISR studies evaluating DCB efficacy and by study feasibility. Analyses were conducted separately for the 1-month and 3-month cohorts. No formal adjustment for multiple comparisons was applied. Because the primary endpoint was not prospectively specified in the original protocol, all findings were interpreted cautiously and were not considered confirmatory. Continuous variables are presented as mean ± standard deviation (SD). Linear mixed-effects models were used to account for within-animal correlation, with animal specified as a random intercept and treatment (LMDCB vs comparator DCB) included as a fixed effect. For repeated measurements, time was included as a fixed effect for QCA (pre-DCB, post-DCB, and follow-up) and for OCT (pre-DCB and follow-up), and between-device comparisons at each time point were derived from these models. All tests were two-sided, and a p-value <0.05 was considered statistically significant.

## Results

### Study population and procedural outcomes

**Fig 2:**
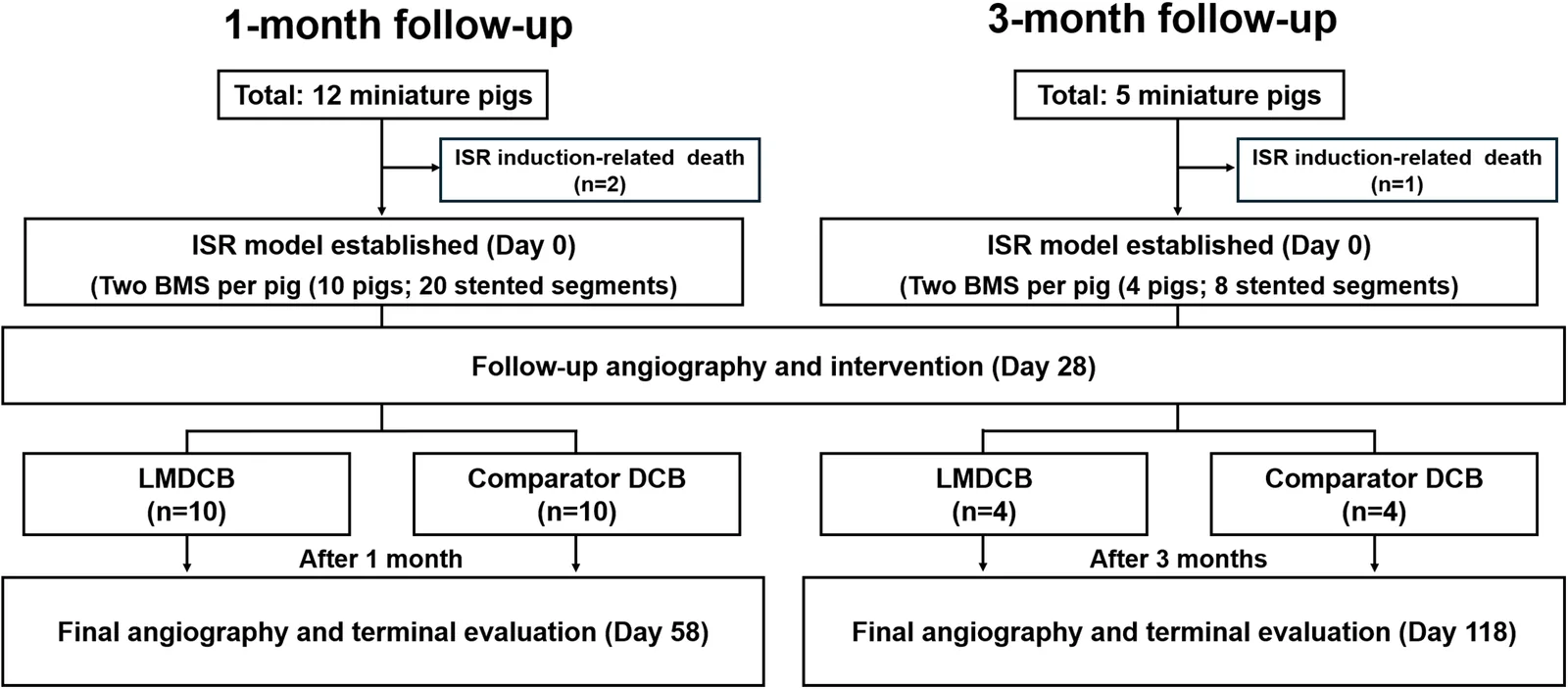
Study flow and cohort design.

Following two procedural deaths during ISR induction in the 1-month cohort and one in the 3-month cohort, the remaining 10 and 4 animals, respectively, underwent DCB treatment and completed the planned terminal follow-up. A total of 14 animals contributed 28 treated coronary segments across the two cohorts: 20 treated segments in the 1-month cohort (10 LMDCB and 10 comparator DCB) and 8 treated segments in the 3-month cohort (4 LMDCB and 4 comparator DCB). The complete study flow, including cohort-specific animal accounting, ISR induction-related deaths, and follow-up assessments, is shown in Fig 2.

ISR induction was attempted in 17 animals: 12 in the 1-month cohort and 5 in the 3-month cohort. Two animals in the 1-month cohort and one animal in the 3-month cohort died during or immediately after ISR induction, before DCB treatment. Accordingly, 10 animals with 20 treated coronary segments underwent 1-month follow-up, and 4 animals with 8 treated coronary segments underwent 3-month follow-up. ISR was induced by balloon injury followed by BMS implantation (two stents per animal) and allowed to develop for 4 weeks. On Day 28, ISR was reassessed by angiography and OCT immediately before DCB treatment. A within-animal paired design was used, with one segment treated with LMDCB and the paired segment treated with the comparator DCB. Final angiography and OCT were performed on Day 58 in the 1-month cohort and on Day 118 in the 3-month cohort, followed by necropsy and histopathological assessment. Histological analysis at 3 months was available for 3 of the 4 animals because of tissue-processing loss. For the LMDCB and comparator DCB groups, n indicates the number of treated coronary segments; animal numbers are reported separately in the cohort flow. ISR, in-stent restenosis; BMS, bare-metal stent; OCT, optical coherence tomography; DCB, drug-coated balloon; LMDCB, linear micro-patterned drug-coated balloon.

In the 3-month cohort, histological analysis was limited to analysable specimens from 3 animals (6 treatment units; 3 per group) due to a technical issue during tissue processing, whereas QCA and OCT analyses were available for all 8 treatment units in this cohort. No device-related complications, including balloon rupture, coating detachment, shaft fracture, or incomplete balloon expansion, occurred during any procedures.

### 1-month outcomes (primary follow-up)

#### QCA.

**Fig 3:**
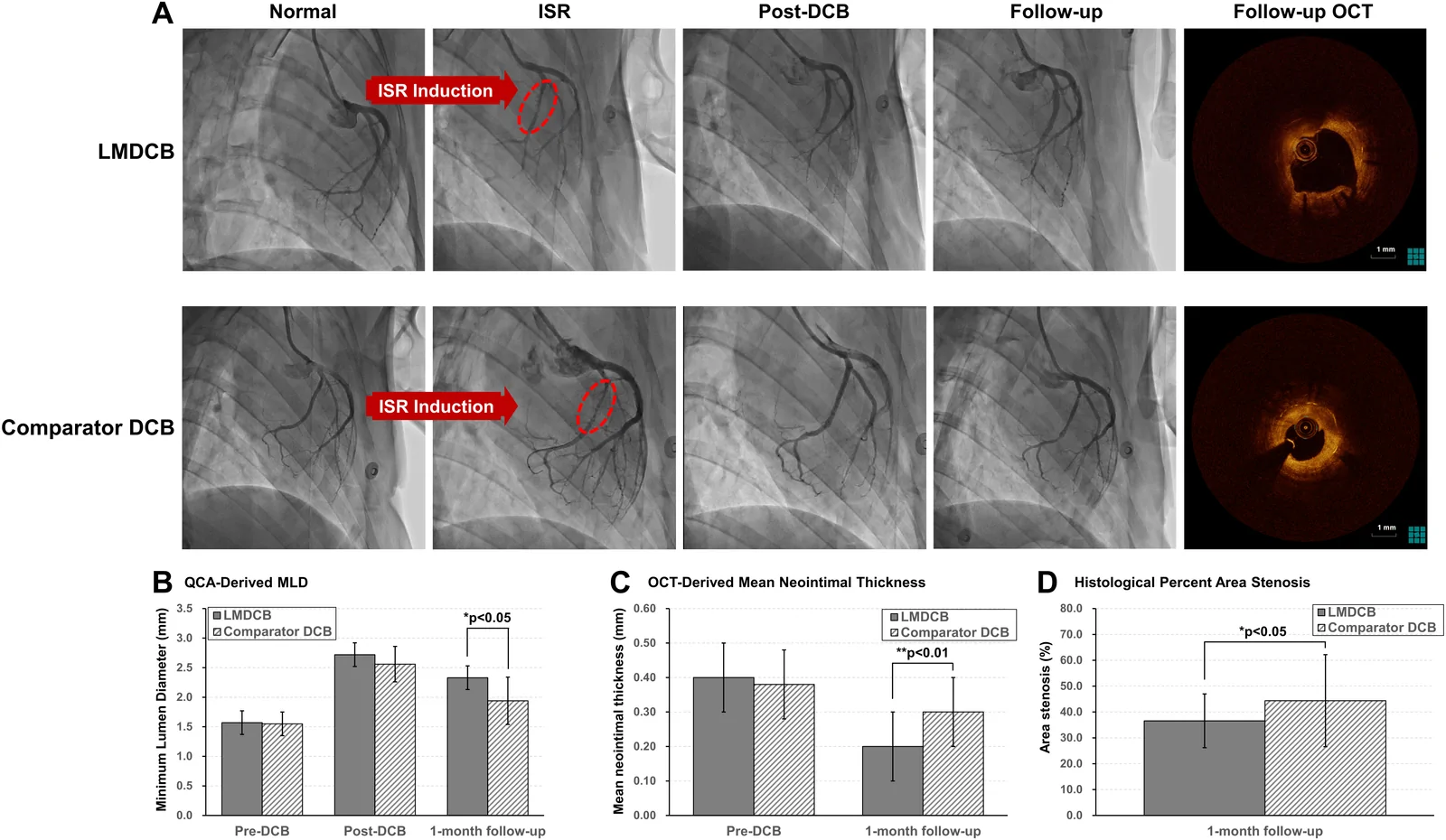
Multimodality assessment of LMDCB versus comparator DCB in a porcine coronary in-stent restenosis model (1-month cohort). (A) Representative coronary angiography and follow-up OCT images from an LMDCB-treated segment (upper row) and a comparator DCB-treated segment (lower row). Sequential angiograms show the normal coronary segment, angiographically confirmed ISR after BMS implantation (red dashed circle; ISR induction), the immediate angiographic result after DCB treatment (post-DCB), and the 1-month follow-up angiogram. Representative OCT cross-sections at 1-month follow-up are shown in the rightmost panels (scale bar, 1 mm). (B) QCA-derived minimum lumen diameter measured at pre-DCB (4 weeks after BMS implantation), immediately after DCB treatment (post-DCB), and at 1-month follow-up. (C) OCT-derived mean neointimal thickness measured at pre-DCB and at 1-month follow-up, with the 1-month measurement designated post hoc as the primary efficacy endpoint for interpretive purposes. (D) Histological percent area stenosis measured at 1-month follow-up. Data are presented as mean ± SD. Statistical comparisons accounted for within-animal correlation. *p < 0.05; **p < 0.01. ISR, in-stent restenosis; DCB, drug-coated balloon; OCT, optical coherence tomography; LMDCB, linear micro-patterned drug-coated balloon; QCA, quantitative coronary angiography; MLD, minimum lumen diameter; BMS, bare-metal stent; SD, standard deviation.

QCA showed no between-group differences at the pre-DCB time point (4 weeks after stenting) or immediately after DCB treatment. Representative angiography and follow-up OCT images are shown in Fig 3A. At 1-month follow-up, LMDCB demonstrated lower percent diameter stenosis than comparator DCB (13.3 ± 4.8% vs 23.7 ± 9.3%; p = 0.003) and a larger minimum lumen diameter (2.3 ± 0.2 mm vs 1.9 ± 0.4 mm; p = 0.019). Acute gain was similar between groups (1.1 ± 0.3 mm vs 1.0 ± 0.3 mm; p = 0.174), whereas late lumen loss was lower with LMDCB (0.4 ± 0.3 mm vs 0.6 ± 0.3 mm; p = 0.027) (Table 2, Fig 3B).

**Table 2:** One-Month Cohort: Complete Timeline (Pre-DCB, Post-DCB, and 1-Month Follow-up).

Parameter	LMDCB (n = 10)	Comparator DCB (n = 10)	p-value
**QCA – Pre-DCB (4 weeks post-stent)**			
Reference diameter (mm)	2.5 ± 0.3	2.7 ± 0.6	0.504
MLD (mm)	1.6 ± 0.2	1.6 ± 0.2	0.850
Percent diameter stenosis (%)	38.3 ± 5.2	41.1 ± 8.1	0.386
**QCA – Post-DCB**			
MLD (mm)	2.7 ± 0.2	2.6 ± 0.3	0.121
Percent diameter stenosis (%)	7.0 ± 2.4	9.2 ± 3.3	0.058
**QCA – 1-Month Follow-up**			
MLD (mm)	2.3 ± 0.2	1.9 ± 0.4	0.019
Percent diameter stenosis (%)	13.3 ± 4.8	23.7 ± 9.3	0.003
**QCA – Changes**			
Acute gain (mm)	1.1 ± 0.3	1.0 ± 0.3	0.174
Late lumen loss (mm)	0.4 ± 0.3	0.6 ± 0.3	0.027
**OCT – Pre-DCB (4 weeks post-stent)**			
Lumen area (mm²)	4.8 ± 1.1	4.5 ± 1.5	0.601
Neointimal area (mm²)	2.3 ± 0.6	2.3 ± 1.0	0.964
Mean neointimal thickness (mm)	0.4 ± 0.1	0.4 ± 0.1	0.623
Percent area stenosis (%)	34.5 ± 10.8	35.3 ± 17.2	0.898
**OCT – 1-Month Follow-up**			
Lumen area (mm²)	6.2 ± 1.1	5.0 ± 1.3	0.061
Neointimal area (mm²)	2.1 ± 0.8	2.5 ± 1.0	0.393
Mean neointimal thickness (mm)	0.2 ± 0.1	0.3 ± 0.1	0.008
Percent area stenosis (%)	25.9 ± 7.2	34.0 ± 14.0	0.121
**Histology – 1-Month Follow-up**			
Lumen area (mm²)	4.2 ± 1.0	3.7 ± 1.3	0.076
Neointimal area (mm²)	2.4 ± 0.7	2.9 ± 1.1	0.011
Percent area stenosis (%)	36.6 ± 10.4	44.4 ± 17.8	0.013
Maximum intimal thickness (mm)	0.4 ± 0.2	0.6 ± 0.2	0.030
Injury score (0–3)	0.3 ± 0.4	0.4 ± 0.5	0.218
Inflammation score (0–3)	0.4 ± 0.5	0.5 ± 0.5	0.275
Endothelialisation score (0–3)	3.0 ± 0.2	2.9 ± 0.3	0.145
Fibrin score	0.2 ± 0.5	0.6 ± 1.0	0.014

Data are presented as mean ± SD. p values were derived from linear mixed-effects models using unrounded data. Some displayed values may appear identical because of rounding, even though the corresponding p values differ. LMDCB, linear micro-patterned drug-coated balloon; DCB, drug-coated balloon; QCA, quantitative coronary angiography; MLD, minimum lumen diameter; OCT, optical coherence tomography; SD, standard deviation.

#### OCT.

OCT parameters were comparable between groups at the pre-DCB time point. At 1 month, the primary efficacy endpoint, OCT-derived mean neointimal thickness, was significantly lower in LMDCB-treated segments than in comparator DCB-treated segments (0.2 ± 0.1 mm vs 0.3 ± 0.1 mm; p = 0.008). LMDCB also showed a numerically larger lumen area (6.2 ± 1.1 mm^2^ vs 5.0 ± 1.3 mm^2^; p = 0.061). Neointimal area (2.1 ± 0.8 mm^2^ vs 2.5 ± 1.0 mm^2^; p = 0.393) and percent area stenosis (25.9 ± 7.2% vs 34.0 ± 14.0%; p = 0.121) did not differ significantly between groups (Table 2, Fig 3C).

### Histology

**Fig 4:**
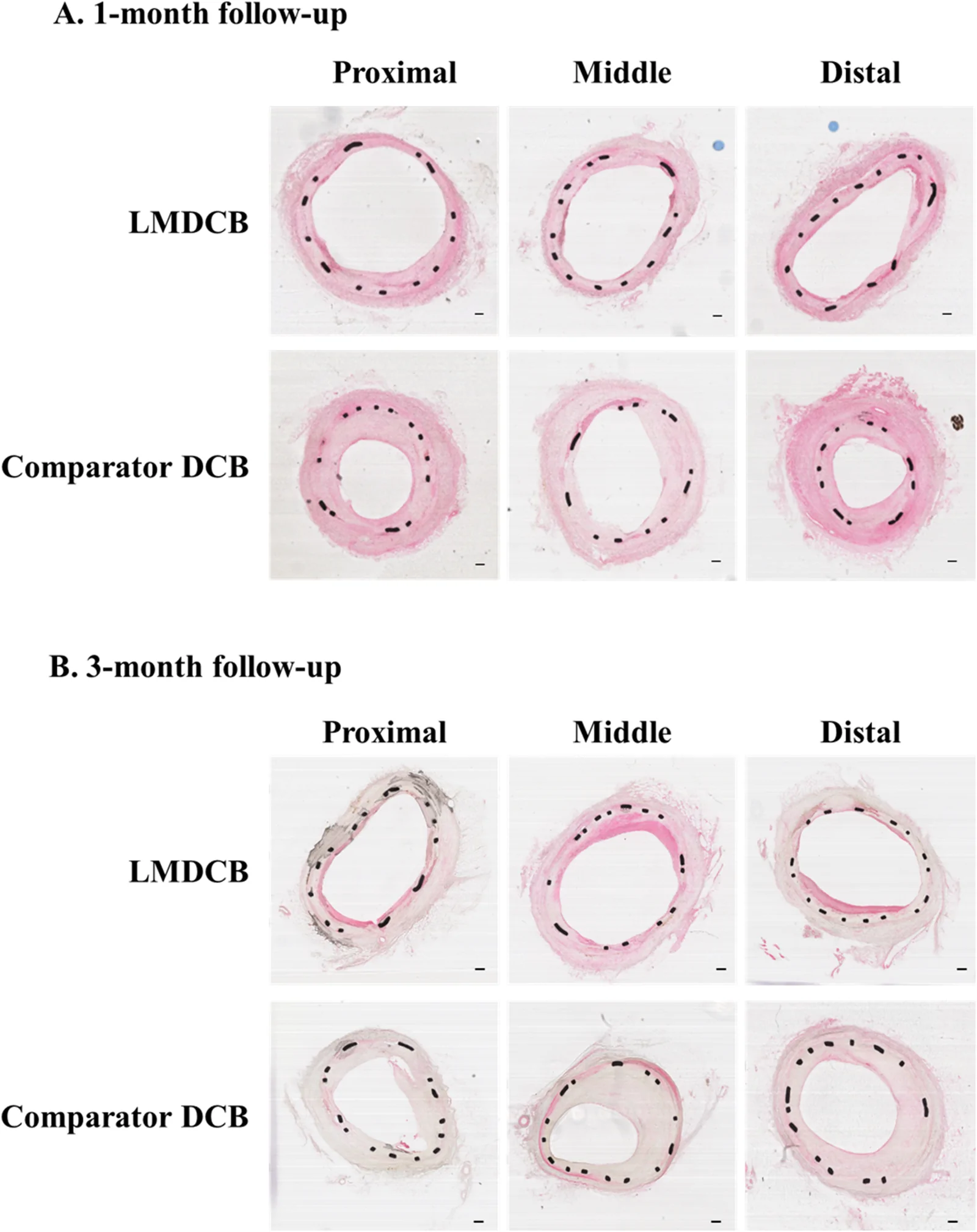
Representative H&E-stained histological sections of LMDCB-treated and comparator DCB-treated segments in the 1- and 3-month cohorts. (A) At 1-month follow-up, representative proximal, middle, and distal cross-sections are shown for LMDCB-treated (top row) and comparator DCB-treated (bottom row) segments. (B) At 3-month follow-up, representative proximal, middle, and distal sections are shown for each group. Sections illustrate peri-strut neointimal formation and associated luminal narrowing. Black markers indicate stent struts. Scale bars, 100 μm. H&E, haematoxylin and eosin; LMDCB, linear micro-patterned drug-coated balloon; DCB, drug-coated balloon.

Histomorphometric analysis at 1 month showed a numerically larger lumen area with LMDCB (4.2 ± 1.0 mm^2^ vs 3.7 ± 1.3 mm^2^; p = 0.076). Neointimal area (2.4 ± 0.7 mm^2^ vs 2.9 ± 1.1 mm^2^; p = 0.011), percent area stenosis (36.6 ± 10.4% vs 44.4 ± 17.8%; p = 0.013), and maximum intimal thickness (0.4 ± 0.2 mm vs 0.6 ± 0.2 mm; p = 0.030) were significantly lower with LMDCB (Table 2, Fig 3D). Healing indices were comparable between groups, including injury score (0.3 ± 0.4 vs 0.4 ± 0.5; p = 0.218), inflammation score (0.4 ± 0.5 vs 0.5 ± 0.5; p = 0.275), and endothelialisation score (3.0 ± 0.2 vs 2.9 ± 0.3; p = 0.145). Fibrin score was lower with LMDCB (0.2 ± 0.5 vs 0.6 ± 1.0; p = 0.014) (Table 2), and representative histological sections are shown in Fig 4A.

### 3-month outcomes (independent cohort)

#### QCA.

QCA showed no between-group differences at the pre-DCB time point or immediately after DCB treatment. At 3 months, QCA parameters were similar between groups, including minimum lumen diameter (2.0 ± 0.7 mm vs 1.8 ± 0.3 mm; p = 0.396), percent diameter stenosis (31.9 ± 6.7% vs 32.5 ± 6.1%; p = 0.550), acute gain (0.6 ± 0.1 mm vs 0.5 ± 0.2 mm; p = 0.537), and late lumen loss (0.5 ± 0.5 mm vs 0.5 ± 0.4 mm; p = 0.809) (Table 3).

**Table 3:** Three-Month Cohort: Complete Timeline (Pre-DCB, Post-DCB, and 3-Month Follow-up).

Parameter	LMDCB (n = 4)	Comparator DCB (n = 4)	p-value
**QCA – Pre-DCB (4 weeks post-stent)**			
Reference diameter (mm)	2.9 ± 0.5	2.9 ± 0.5	0.923
MLD (mm)	2.0 ± 0.4	1.8 ± 0.4	0.477
Percent diameter stenosis (%)	32.5 ± 2.2	36.5 ± 7.5	0.308
**QCA – Post-DCB**			
MLD (mm)	2.5 ± 0.4	2.3 ± 0.2	0.370
Percent diameter stenosis (%)	14.9 ± 2.9	13.0 ± 0.6	0.261
**QCA – 3-Month Follow-up**			
MLD (mm)	2.0 ± 0.7	1.8 ± 0.3	0.396
Percent diameter stenosis (%)	31.9 ± 6.7	32.5 ± 6.1	0.550
**QCA – Changes**			
Acute gain (mm)	0.6 ± 0.1	0.5 ± 0.2	0.537
Late lumen loss (mm)	0.5 ± 0.5	0.5 ± 0.4	0.809
**OCT – Pre-DCB (4 weeks post-stent)**			
Lumen area (mm²)	5.1 ± 1.8	5.1 ± 1.2	0.941
Neointimal area (mm²)	2.9 ± 0.5	3.3 ± 0.9	0.508
Mean neointimal thickness (mm)	0.3 ± 0.1	0.4 ± 0.1	0.613
Percent area stenosis (%)	37.8 ± 12.0	39.7 ± 10.2	0.775
**OCT – 3-Month Follow-up**			
Lumen area (mm²)	5.4 ± 3.4	5.3 ± 1.3	0.967
Neointimal area (mm²)	3.3 ± 1.5	3.5 ± 0.6	0.800
Mean neointimal thickness (mm)	0.4 ± 0.3	0.4 ± 0.1	0.908
Percent area stenosis (%)	42.1 ± 25.7	40.3 ± 9.5	0.890
**Histology – 3-Month Follow-up (n = 3)**			
Lumen area (mm²)	5.0 ± 1.1	3.7 ± 1.4	0.041
Neointimal area (mm²)	2.4 ± 0.5	3.1 ± 1.0	0.065
Percent area stenosis (%)	32.9 ± 8.9	46.4 ± 10.9	0.011
Maximum intimal thickness (mm)	0.5 ± 0.3	0.7 ± 0.3	0.050
Injury score (0–3)	0.2 ± 0.4	0.3 ± 0.5	0.625
Inflammation score (0–3)	0.2 ± 0.4	0.3 ± 0.5	0.625
Endothelialisation score (0–3)	2.9 ± 0.3	2.9 ± 0.3	1.000
Fibrin score	0.1 ± 0.3	0.2 ± 0.4	0.475

Data are presented as mean ± SD. p values were derived from linear mixed-effects models using unrounded data. Some displayed values may appear identical because of rounding, even though the corresponding p values differ. p values are displayed to 3 decimal places. The unrounded p value for maximum intimal thickness was 0.0497 and is displayed as 0.050 after rounding to 3 decimal places. LMDCB, linear micro-patterned drug-coated balloon; DCB, drug-coated balloon; QCA, quantitative coronary angiography; MLD, minimum lumen diameter; OCT, optical coherence tomography; SD, standard deviation. Histology at 3-month follow-up was available in 3 animals per group because of tissue-processing loss.

#### OCT.

OCT parameters showed no between-group differences at the pre-DCB time point. At 3 months, OCT parameters were also comparable between groups, including lumen area (5.4 ± 3.4 mm² vs 5.3 ± 1.3 mm²; p = 0.967), neointimal area (3.3 ± 1.5 mm² vs 3.5 ± 0.6 mm²; p = 0.800), mean neointimal thickness (0.4 ± 0.3 mm vs 0.4 ± 0.1 mm; p = 0.908), and percent area stenosis (42.1 ± 25.7% vs 40.3 ± 9.5%; p = 0.890) (Table 3).

### Histology

**Fig 5:**
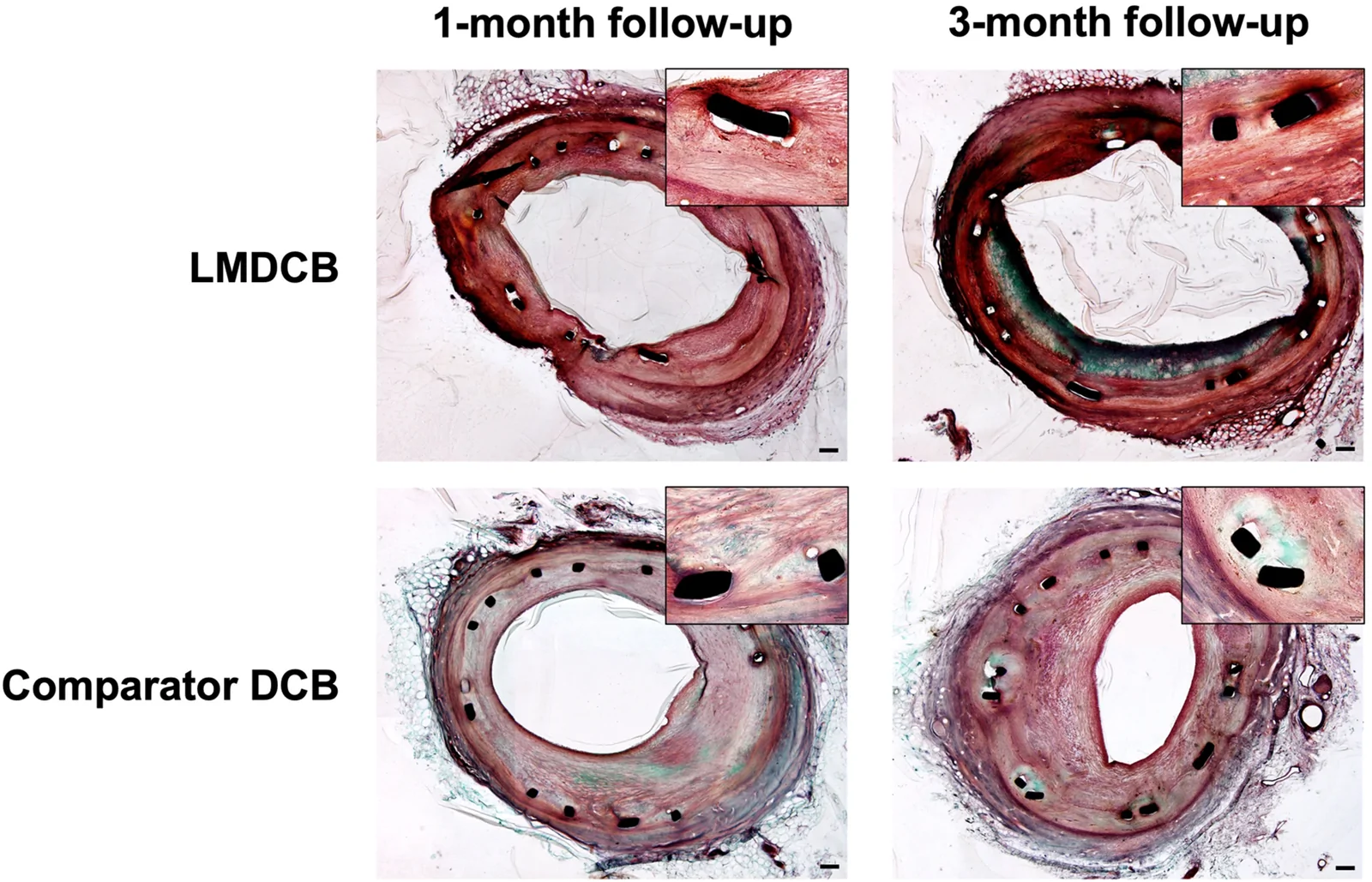
Representative Masson’s trichrome-stained histological sections of LMDCB-treated and comparator DCB-treated segments in the 1- and 3-month cohorts. Representative cross-sections are shown from LMDCB-treated segments (upper row) and comparator DCB-treated segments (lower row) at 1-month (left column) and 3-month (right column) follow-up. Sections illustrate neointimal tissue formation with associated luminal narrowing. Trichrome staining highlights collagen-rich extracellular matrix (blue/green) and cellular components including smooth muscle/cytoplasm (red), allowing qualitative assessment of neointimal composition across time points. Insets show higher-magnification views of peri-strut regions (stent strut voids). Scale bars, 100 μm. LMDCB, linear micro-patterned drug-coated balloon; DCB, drug-coated balloon.

Histological analysis at 3 months was available in a subset of 3 animals (6 treatment units; 3 per group). Within this subset, lumen area was larger with LMDCB (5.0 ± 1.1 mm² vs 3.7 ± 1.4 mm²; p = 0.041) and percent area stenosis was lower (32.9 ± 8.9% vs 46.4 ± 10.9%; p = 0.011). Neointimal area was numerically lower with LMDCB (2.4 ± 0.5 mm² vs 3.1 ± 1.0 mm²; p = 0.065), while maximum intimal thickness was significantly lower (0.5 ± 0.3 mm vs 0.7 ± 0.3 mm; p = 0.0497) (Table 3). Healing indices were similar between groups, including injury, inflammation, endothelialisation, and fibrin scores (Table 3). Representative histological sections are shown in Fig 4B, and additional Masson’s trichrome-stained sections at 1 and 3 months are provided in Fig 5.

## Discussion

This preclinical study evaluated a surface-engineered drug-coated balloon (LMDCB) compared with a clinically used comparator paclitaxel-coated DCB in a staged porcine coronary ISR model. In the 1-month cohort, LMDCB was associated with improved luminal dimensions, with lower percent diameter stenosis (13.3 ± 4.8% vs 23.7 ± 9.3%; p = 0.003) and larger minimum lumen diameter (2.3 ± 0.2 mm vs 1.9 ± 0.4 mm; p = 0.019) compared with the comparator DCB. These findings were supported by reduced mean neointimal thickness on OCT (0.2 ± 0.1 mm vs 0.3 ± 0.1 mm; p = 0.008) and lower percent area stenosis on histomorphometry (36.6 ± 10.4% vs 44.4 ± 17.8%; p = 0.013). In the independent 3-month cohort, angiographic and OCT outcomes were similar between devices, while exploratory histomorphometry in a limited subset suggested a larger lumen area and lower percent area stenosis with LMDCB. Vascular healing indices were comparable between groups, and no device-related complications were observed.

The therapeutic performance of DCBs depends on efficient transfer of the antiproliferative drug during a brief balloon inflation period, typically lasting 30–60 seconds. Unlike drug-eluting stents, which provide sustained drug release over weeks to months, DCBs rely on rapid contact-mediated transfer during a single balloon inflation [14]. In ISR lesions, heterogeneous neointimal morphology, irregular luminal geometry, and variable tissue compliance may impair uniform balloon apposition, potentially leading to spatial variability in drug transfer even when angiographic sizing appears appropriate [15,16]. These considerations suggest that DCB efficacy depends not only on the drug itself but also on the quality of balloon–artery contact achieved during inflation.

Clinical and real-world data demonstrate considerable heterogeneity in outcomes after DCB treatment of ISR, and differences in late lumen loss and target lesion revascularisation have been reported across lesion subsets and device platforms [17,18]. Importantly, head-to-head comparisons of paclitaxel-, sirolimus-, and biolimus-coated balloons have not consistently demonstrated superiority of a specific drug class [19–21]. A recent network meta-analysis likewise suggested endpoint-dependent rankings rather than a uniformly superior DCB platform [22]. Even within paclitaxel-based devices, excipient chemistry, coating crystallinity, and arterial transfer and retention characteristics may produce device-specific biological responses, indicating that delivery mechanics influence efficacy beyond nominal drug identity alone [23–25]. In the present study, both devices used paclitaxel at the same nominal drug dose density of 3.0 µg/mm² and had identical balloon dimensions of 3.0 × 20 mm; however, they differed in coating matrix, balloon surface architecture, and manufacturer-specified nominal pressure. The LMDCB used a shellac-based natural-resin matrix and was inflated at its nominal pressure of 8 atm, whereas the comparator DCB used a BTHC-based matrix and was inflated at its nominal pressure of 7 atm. Excipient chemistry, coating characteristics, surface architecture, and nominal inflation pressure may each influence balloon–artery interaction, drug release, arterial transfer, and tissue retention. Because the present study compared complete device platforms rather than otherwise identical balloons differing only in surface architecture, the independent contribution of surface micro-patterning could not be distinguished from the effects of coating chemistry, nominal inflation pressure, and other device-level characteristics. Accordingly, the observed biological differences cannot be attributed solely to the micro-patterned surface.

The micro-patterned platform evaluated in this study was designed to address apposition variability by enhancing focal contact engagement during balloon inflation, which may facilitate drug transfer to the vessel wall. Prior preclinical evaluation of the same platform demonstrated approximately eightfold higher maximum contact pressure and 2.3-fold greater retained tissue paclitaxel at 48 hours in rabbit iliac arteries than a conventional flat-surfaced DCB [10]. These findings were obtained in prior platform-characterisation work using a smaller prototype configuration and were not directly re-evaluated using the 3.0 × 20 mm device configuration assessed in the present study. Independent external validation specifically linking surface micro-patterning to increased contact pressure and arterial drug transfer remains limited. The same study also reported favourable QCA and OCT findings in a minipig coronary ISR model, although coronary tissue pharmacokinetics were not assessed. Because the comparator used in that study differed from the Pantera Lux DCB evaluated in the present study, these findings do not provide a direct pharmacokinetic comparison between the two devices. Enhanced drug transfer therefore remains a mechanistically plausible but unconfirmed explanation for the lower neointimal response observed at 1 month, as arterial paclitaxel concentrations were not measured in the present study. Differences in mechanical injury, balloon compliance, or contact-pressure distribution between the two device platforms may also have contributed to the observed 1-month findings, and the relative contributions of these factors and enhanced drug transfer cannot be distinguished from the available data. Importantly, the between-device differences observed at 1 month occurred despite similar acute angiographic results immediately after the procedure, suggesting that the separation between treatment groups primarily reflected differences in subsequent late lumen loss rather than immediate procedural effects. Histological findings showed reduced neointimal formation without evidence of increased injury or inflammation, with no evident adverse histological vascular response observed for the micro-patterned device in this model. The lower fibrin score observed with LMDCB at 1 month was compatible with the absence of delayed vascular healing, whereas injury, inflammation, and endothelialisation scores were comparable between groups. Because arterial paclitaxel concentrations and coagulation-related tissue markers were not measured, whether this finding reflected differences in local paclitaxel exposure or in the coagulation micro-environment could not be determined. However, because fibrin deposition was assessed semi-quantitatively in a limited cohort and no formal adjustment for multiple comparisons was applied, this isolated finding should be regarded as exploratory and should not be interpreted as evidence of superior vascular healing. The discrepancy in statistical significance between OCT and histological findings for neointimal area and percent area stenosis at 1 month may reflect differences in sampling density and anatomical correspondence between the two modalities. OCT measurements were obtained at 1-mm intervals throughout the stented segment, whereas histological assessment was based on selected proximal, middle, and distal sections. In addition, fixation, sectioning, and resin embedding may have altered tissue dimensions and contributed to differences between in vivo OCT and ex vivo histological measurements. Direct section-matched registration between OCT and histology was not performed at either follow-up time point.

The independent 3-month cohort was included to provide supportive mid-term context for vascular response over time. At this time point, angiographic and OCT outcomes were similar between LMDCB and comparator DCB, which may partly reflect limited statistical power in a small cohort (n = 4 treatment units per group) to detect modest between-group differences. In addition, paclitaxel’s antiproliferative effects are most pronounced during the early period after delivery, and early differences in local drug exposure, if present, may attenuate over time as vascular remodelling progresses. Given that the comparator was a clinically used paclitaxel-coated DCB platform, both devices may have achieved sufficient antiproliferative effect to yield similar mid-term imaging outcomes in this model. Histological assessment at 3 months was further constrained by tissue-processing issues that reduced the analysable subset, and histological findings at this time point should therefore be interpreted as exploratory rather than confirmatory evidence of sustained efficacy.

Several limitations should be acknowledged. The sample size was relatively small, particularly in the independent 3-month cohort, limiting statistical power to detect modest between-group differences. Because the 1-month and 3-month cohorts were conducted sequentially in separate animals and analysed independently, direct longitudinal comparison of vascular responses within the same animals was not possible. The findings from the smaller 3-month cohort should therefore be interpreted as supportive and exploratory rather than confirmatory. Because detailed anatomical, procedural, and animal-level data were insufficient to permit a reliable comparison between animals that died during ISR induction and those that survived, the possibility of unrecognised survival-related selection cannot be excluded. In addition, because a single primary efficacy endpoint was not prospectively specified in the original protocol and no formal adjustment for multiple comparisons was applied, the study findings should be interpreted cautiously and should not be considered confirmatory. Follow-up was limited to 1 and 3 months and therefore does not provide information regarding long-term durability or late safety. No untreated ISR control group was included, limiting assessment of the natural progression of ISR in this model. Because the present study compared complete device platforms rather than otherwise identical balloons differing only in surface architecture, the independent contribution of surface micro-patterning cannot be separated from the effects of coating chemistry, nominal inflation pressure, and other device-level characteristics. Accordingly, the observed biological differences cannot be attributed solely to the micro-patterned surface. Although treatment allocation remained concealed until the paired target segments had been selected and baseline angiographic and OCT assessments had been completed, the interventional operator could not be blinded during device preparation and deployment; therefore, performance bias related to operator-dependent procedural decisions cannot be completely excluded. Direct arterial pharmacokinetic measurements were not performed, and enhanced drug transfer therefore remains a mechanistically plausible but unconfirmed explanation for the observed 1-month findings. Finally, the porcine ISR model does not fully replicate the biological and mechanical complexity of human ISR, including neoatherosclerosis, mechanical stent factors, and heterogeneous inflammatory responses [26,27].

Despite these limitations, the present study provides proof-of-concept evidence of favourable early biological performance of the LMDCB in a preclinical ISR model without evidence of impaired vascular healing. Translation of these findings will require staged validation in larger cohorts with extended follow-up to assess long-term vascular healing and durability. To date, the investigational LMDCB evaluated in the present study has not been assessed in humans. Accordingly, future first-in-human evaluation is needed to establish its safety and feasibility and to determine whether the preclinical findings observed in this study translate into clinically relevant effects.

## Conclusion

In this staged preclinical porcine coronary ISR study, LMDCB was associated with reduced neointimal proliferation and improved luminal dimensions at 1 month compared with a comparator paclitaxel-coated DCB, without evidence of impaired vascular healing. Other luminal measures at 1 month were not consistently statistically significant. In an independent 3-month cohort, angiographic and OCT outcomes were similar between devices, while exploratory histology provided limited supportive evidence for luminal preservation at the later follow-up time point. These findings should be interpreted within the constraints of a porcine ISR model established in otherwise healthy animals, which does not reproduce the neoatherosclerotic, calcific, and lipid-rich substrates of human ISR and may differ from clinical lesions in balloon–vessel wall contact and apposition. Further studies incorporating longer follow-up, direct assessment of arterial drug transfer, and appropriately matched device comparisons are needed to strengthen the mechanistic evidence and support clinical translation.

## Supporting information

S1 Dataset Complete dataset. This file contains all data analysed during this study.
(XLSX)

## References

[1] Alfonso F, Byrne RA, Rivero F, Kastrati A. Current treatment of in-stent restenosis. J Am Coll Cardiol. 2014;63(24):2659–73. doi: 10.1016/j.jacc.2014.02.545 24632282

[2] Neumann F-J, Sousa-Uva M, Ahlsson A, Alfonso F, Banning AP, Benedetto U, et al. 2018 ESC/EACTS Guidelines on myocardial revascularization. Eur Heart J. 2019;40(2):87–165. doi: 10.1093/eurheartj/ehy394 30165437

[3] Scheller B, Hehrlein C, Bocksch W, Rutsch W, Haghi D, Dietz U, et al. Treatment of coronary in-stent restenosis with a paclitaxel-coated balloon catheter. N Engl J Med. 2006;355(20):2113–24. doi: 10.1056/NEJMoa061254 17101615

[4] Jeger RV, Eccleshall S, Wan Ahmad WA, Ge J, Poerner TC, Shin ES, et al. Drug-Coated Balloons for Coronary Artery Disease: Third Report of the International DCB Consensus Group. JACC Cardiovasc Interv. 2020;13:1391–402. doi: 10.1016/j.jcin.2020.02.043 32473887

[5] Tanaka A, Latib A, Jabbour RJ, Kawamoto H, Giannini F, Ancona M, et al. Impact of Angiographic Result After Predilatation on Outcome After Drug-Coated Balloon Treatment of In-Stent Coronary Restenosis. Am J Cardiol. 2016;118(10):1460–5. doi: 10.1016/j.amjcard.2016.08.006 27634028

[6] Rhee T-M, Lee JM, Shin E-S, Hwang D, Park J, Jeon K-H, et al. Impact of Optimized Procedure-Related Factors in Drug-Eluting Balloon Angioplasty for Treatment of In-Stent Restenosis. JACC Cardiovasc Interv. 2018;11(10):969–78. doi: 10.1016/j.jcin.2018.02.002 29798774

[7] Scheller B, Mangner N, Abdul Kader MASK, Wan Ahmad WA, Jeger R, Wöhrle J, et al. Combined Analysis of Two Parallel Randomized Trials of Sirolimus-Coated and Paclitaxel-Coated Balloons in Coronary In-Stent Restenosis Lesions. Circ Cardiovasc Interv. 2022;15(9):e012305. doi: 10.1161/CIRCINTERVENTIONS.122.012305 36126132

[8] Hu P, Sun Y, Li C-L, Jin R, Xie Q, Jiang X-J, et al. A randomized comparison of two paclitaxel-coated balloons for the treatment of in-stent restenosis: The LONGTY ISR China randomized trial (LONGTY DCB vs. SeQuent Please DCB). Catheter Cardiovasc Interv. 2021;97 Suppl 2:988–95. doi: 10.1002/ccd.29589 33734575

[9] Granada JF, Stenoien M, Buszman PP, Tellez A, Langanki D, Kaluza GL, et al. Mechanisms of tissue uptake and retention of paclitaxel-coated balloons: impact on neointimal proliferation and healing. Open Heart. 2014;1(1):e000117. doi: 10.1136/openhrt-2014-000117 25332821 PMC4189287

[10] Lee K, Lee SG, Jang I, Park SH, Yang D, Seo IH, et al. Linear Micro-patterned Drug Eluting Balloon (LMDEB) for Enhanced Endovascular Drug Delivery. Sci Rep. 2018;8(1):3666. doi: 10.1038/s41598-018-21649-7 29507314 PMC5838243

[11] Schwartz RS, Huber KC, Murphy JG, Edwards WD, Camrud AR, Vlietstra RE, et al. Restenosis and the proportional neointimal response to coronary artery injury: results in a porcine model. J Am Coll Cardiol. 1992;19(2):267–74. doi: 10.1016/0735-1097(92)90476-4 1732351

[12] Lim SY, Jeong MH, Hong SJ, Lim DS, Moon JY, Hong YJ, et al. Inflammation and delayed endothelization with overlapping drug-eluting stents in a porcine model of in-stent restenosis. Circ J. 2008;72(3):463–8. doi: 10.1253/circj.72.463 18296847

[13] Joner M, Byrne RA, Lapointe J-M, Radke PW, Bayer G, Steigerwald K, et al. Comparative assessment of drug-eluting balloons in an advanced porcine model of coronary restenosis. Thromb Haemost. 2011;105(5):864–72. doi: 10.1160/TH10-11-0698 21301785

[14] Scheller B, Speck U, Abramjuk C, Bernhardt U, Böhm M, Nickenig G. Paclitaxel balloon coating, a novel method for prevention and therapy of restenosis. Circulation. 2004;110(7):810–4. doi: 10.1161/01.CIR.0000138929.71660.E0 15302790

[15] Byrne RA, Joner M, Kastrati A. Stent thrombosis and restenosis: what have we learned and where are we going? The Andreas Grüntzig Lecture ESC 2014. Eur Heart J. 2015;36:3320–31. doi: 10.1093/eurheartj/ehv511 PMC4677274 26417060

[16] Yerasi C, Case BC, Forrestal BJ, Torguson R, Weintraub WS, Garcia-Garcia HM, et al. Drug-Coated Balloon for De Novo Coronary Artery Disease: JACC State-of-the-Art Review. J Am Coll Cardiol. 2020;75:1061–73. doi: 10.1016/j.jacc.2019.12.046 32138967

[17] Giacoppo D, Alfonso F, Xu B, Claessen BEPM, Adriaenssens T, Jensen C, et al. Paclitaxel-coated balloon angioplasty vs. drug-eluting stenting for the treatment of coronary in-stent restenosis: a comprehensive, collaborative, individual patient data meta-analysis of 10 randomized clinical trials (DAEDALUS study). Eur Heart J. 2020;41(38):3715–28. doi: 10.1093/eurheartj/ehz594 31511862 PMC7706792

[18] Bondesson P, Lagerqvist B, James SK, Olivecrona GK, Venetsanos D, Harnek J. Comparison of two drug-eluting balloons: a report from the SCAAR registry. EuroIntervention. 2012;8(4):444–9. doi: 10.4244/EIJV8I4A70 22917727

[19] Ali RM, Abdul Kader MASK, Wan Ahmad WA, Ong TK, Liew HB, Omar A-F, et al. Treatment of Coronary Drug-Eluting Stent Restenosis by a Sirolimus- or Paclitaxel-Coated Balloon. JACC Cardiovasc Interv. 2019;12(6):558–66. doi: 10.1016/j.jcin.2018.11.040 30898253

[20] Chen Y, Gao L, Qin Q, Zhang J, Jia S, Wu M, et al. Biolimus-coated versus paclitaxel-coated balloons for coronary in-stent restenosis (BIO ASCEND ISR): a randomised, non-inferiority trial. EuroIntervention. 2024;20(13):e806–17. doi: 10.4244/EIJ-D-24-00295 38742581 PMC11200665

[21] Liu H, Li Y, Fu G, An J, Chen S, Zhong Z, et al. Sirolimus- vs Paclitaxel-Coated Balloon for the Treatment of Coronary In-Stent Restenosis: The SIBLINT-ISR Randomized Trial. JACC Cardiovasc Interv. 2025;18:963–71. doi: 10.1016/j.jcin.2024.12.024 39985511

[22] Guo S, Bi C, Wang X, Lv T, Zhang Z, Chen X, et al. Comparative efficacy of interventional therapies and devices for coronary in-stent restenosis: A systematic review and network meta-analysis of randomized controlled trials. Heliyon. 2024;10(6):e27521. doi: 10.1016/j.heliyon.2024.e27521 38496861 PMC10944233

[23] Colleran R, Joner M, Kufner S, Altevogt F, Neumann F-J, Abdel-Wahab M, et al. Comparative efficacy of two paclitaxel-coated balloons with different excipient coatings in patients with coronary in-stent restenosis: A pooled analysis of the Intracoronary Stenting and Angiographic Results: Optimizing Treatment of Drug Eluting Stent In-Stent Restenosis 3 and 4 (ISAR-DESIRE 3 and ISAR-DESIRE 4) trials. Int J Cardiol. 2018;252:57–62. doi: 10.1016/j.ijcard.2017.11.076 29203209

[24] Tzafriri AR, Muraj B, Garcia-Polite F, Salazar-Martín AG, Markham P, Zani B, et al. Balloon-based drug coating delivery to the artery wall is dictated by coating micro-morphology and angioplasty pressure gradients. Biomaterials. 2020;260:120337. doi: 10.1016/j.biomaterials.2020.120337 32937269 PMC7530113

[25] Kempin W, Kaule S, Reske T, Grabow N, Petersen S, Nagel S, et al. In vitro evaluation of paclitaxel coatings for delivery via drug-coated balloons. Eur J Pharm Biopharm. 2015;96:322–8. doi: 10.1016/j.ejpb.2015.08.010 26318979

[26] Lowe HC, Schwartz RS, Mac Neill BD, Jang I-K, Hayase M, Rogers C, et al. The porcine coronary model of in-stent restenosis: current status in the era of drug-eluting stents. Catheter Cardiovasc Interv. 2003;60(4):515–23. doi: 10.1002/ccd.10705 14624433

[27] Giustino G, Colombo A, Camaj A, Yasumura K, Mehran R, Stone GW, et al. Coronary In-Stent Restenosis: JACC State-of-the-Art Review. J Am Coll Cardiol. 2022;80(4):348–72. doi: 10.1016/j.jacc.2022.05.017 35863852

